# Case Report: Novel SAVI-Causing Variants in *STING1* Expand the Clinical Disease Spectrum and Suggest a Refined Model of STING Activation

**DOI:** 10.3389/fimmu.2021.636225

**Published:** 2021-03-22

**Authors:** Bin Lin, Sofia Torreggiani, Dana Kahle, Dax G. Rumsey, Benjamin L. Wright, Marco A. Montes-Cano, Laura Fernandez Silveira, Sara Alehashemi, Jacob Mitchell, Alexander G. Aue, Zheng Ji, Tengchuan Jin, Adriana A. de Jesus, Raphaela Goldbach-Mansky

**Affiliations:** ^1^ Laboratory of Clinical Immunology and Microbiology, Translational Autoinflammatory Diseases Section (TADS), National Institute of Allergy and Infectious Diseases, National Institutes of Health, Bethesda, MD, United States; ^2^ Department of Pediatrics, University of Alberta, Edmonton, AB, Canada; ^3^ Section of Allergy and Immunology, Division of Pulmonology, Phoenix Children’s Hospital, Phoenix, AZ, United States; ^4^ Division of Allergy, Asthma, and Clinical Immunology, Department of Medicine, Mayo Clinic, Scottsdale, AZ, United States; ^5^ Immunology Service, Hospital Universitario Virgen del Rocío, Seville, Spain; ^6^ Pediatric Immunodeficiency, Rheumatology and Infectious Diseases Unit, Hospital Universitario Virgen del Rocío, Seville, Spain; ^7^ Laboratory of Structural Immunology, CAS Key Laboratory of Innate Immunity and Chronic Disease, CAS Center for Excellence in Molecular Cell Science, School of Life Sciences and Medical Center, University of Science and Technology of China, Hefei, China

**Keywords:** interferonopathy, autoinflammatory disease, type I interferon, SAVI, STING, whole exome sequencing, pediatrics

## Abstract

Gain-of-function mutations in *STING1* cause the monogenic interferonopathy, SAVI, which presents with early-onset systemic inflammation, cold-induced vasculopathy and/or interstitial lung disease. We identified 5 patients (3 kindreds) with predominantly peripheral vascular disease who harbor 3 novel *STING1* variants, p.H72N, p.F153V, and p.G158A. The latter two were predicted by a previous cryo-EM structure model to cause STING autoactivation. The p.H72N variant in exon 3, however, is the first SAVI-causing variant in the transmembrane linker region. Mutations of p.H72 into either charged residues or hydrophobic residues all led to dramatic loss of cGAMP response, while amino acid changes to residues with polar side chains were able to maintain the wild type status. Structural modeling of these novel mutations suggests a reconciled model of STING activation, which indicates that STING dimers can oligomerize in both open and closed states which would obliviate a high-energy 180° rotation of the ligand-binding head for STING activation, thus refining existing models of STING activation. Quantitative comparison showed that an overall lower autoactivating potential of the disease-causing mutations was associated with less severe lung disease, more severe peripheral vascular disease and the absence of a robust interferon signature in whole blood. Our findings are important in understanding genotype-phenotype correlation, designing targeted STING inhibitors and in dissecting differentially activated pathways downstream of different STING mutations.

## Introduction

Autoactivating variants in Stimulator of interferon response cGAMP interactor 1 (*STING1*, also known as *TMEM173*), the gene that encodes STING (Stimulator of IFN genes) (1–3) cause a rare autoinflammatory interferonopathy, STING-associated vasculopathy with onset in infancy (SAVI, OMIM # 615934) (4–6). SAVI-causing variants in exons 5, 6 and 7 were so far found in 8 different amino acid residues that lead to STING autoactivation in the absence of ligand-binding (5–11). Here we report 3 novel SAVI-causing mutations in 3 unrelated kindreds, including variant p.H72N, which was disease-causing in a mother and 2 children, and is the first disease-causing gain-of-function (GOF) mutation in the transmembrane linker region of STING (12). Modeling of previously reported and these novel SAVI mutations extend our current understanding of STING activation involving critical residues that are mutated in the connector helix loop encoded by exon 5, the polymer interface encoded by exon 6 and 7, and the transmembrane linker region encoded by exon 3.

## Materials and Methods

### *IFNB1* Luciferase Reporter Assay

The assay was performed as previously described in detail (13). Briefly, HEK293T cells were co-transfected with STING constructs and *IFNB1* firefly luciferase reporter construct with Lipofectamine 3000 reagent (ThermoFisher Scientific, cat# L3000015) in 96-well plates (black wall with clear bottom, BD Falcon, cat# 353219) with a reverse transfection protocol. 50 pg of STING construct and 50 ng of *IFNB1* firefly luciferase reporter construct were mixed with 5 μL of Opti-MEM and 0.2 μL of P300 reagent. The vector dilutions were then mixed with the Lipofectamine 3000 dilutions containing 5 μL of Opti-MEM and 0.3 μL of Lipofectamine 3000, and then incubated at room temperature for 10-30 min. 30,000 HEK293T cells in 75 μL of complete DMEM were applied to each well and mixed by gently tapping, and then rocked back and forth to allow even distribution of the cells, followed by 10 min incubation at room temperature to allow the cells to settle. Plates were then returned to 37°C in a humidified atmosphere with 5% CO2 for 24 hr. Luciferase assay was carried out using ONE-Glo™ EX Luciferase Assay System (Promega, cat# E8120), with 50 μL luciferase reagent per well.

### cGAMP Stimulation

1.8 μL of 2’3’-cGAMP (1 μg/μL, Invivogene, cat# tlrl-nacga23) were diluted with 3.2 μL of complete DMEM, and applied to one well (96-well plate) with 85 μL of culture at 6 hr post transfection for a final concentration of 20 μg/mL (13). For the 4 μg/mL dose, 0.36 μL of 2’3’-cGAMP were diluted with 4.64 μL of complete DMEM.

### Western Blot

Transfections were carried out as described above, except 24-well plates were used (Corning Costar, cat# 3524) and reagents and cells were added in quantities 4-times as in the 96-well plate. Briefly, 800 pg of STING construct and 200 ng of *IFNB1* firefly luciferase reporter construct were co-transfected into 120,000 HEK293T cells in each well.

Western blot was carried out as previously described (13). Briefly, 12 μg of protein were loaded for each sample and transferred to PVDF membranes (Trans-Blot^®^ Turbo™ Midi PVDF Transfer Packs, BIO-RAD, cat# 1704157) with Trans-Blot^®^ Turbo™ Transfer System (BIO-RAD, cat#1704150). STING and ACTB were detected simultaneously by 0.2 µg/mL of anti-STING antibody (R&D Systems, cat# MAB7169-SP) and hFAB™ Rhodamine Anti-Actin Primary Antibody (BIO-RAD, cat# 12004163, 1:1,000 dilution) under chemiluminescent and rhodamine channels, respectively.

### Modeling

The side chain of p.H72 residue in the original 4.1 Å resolution of full-length human STING cryo-electron microscopy (Cryo-EM) structure (pdb 6NT5) was flipped and the energy of the structure was minimized by MOE (Molecular Operating Environment) software, resulting in a stereochemically more favorable conformation of p.H72.

## Results

### Clinical Presentations

**Figure 1:**
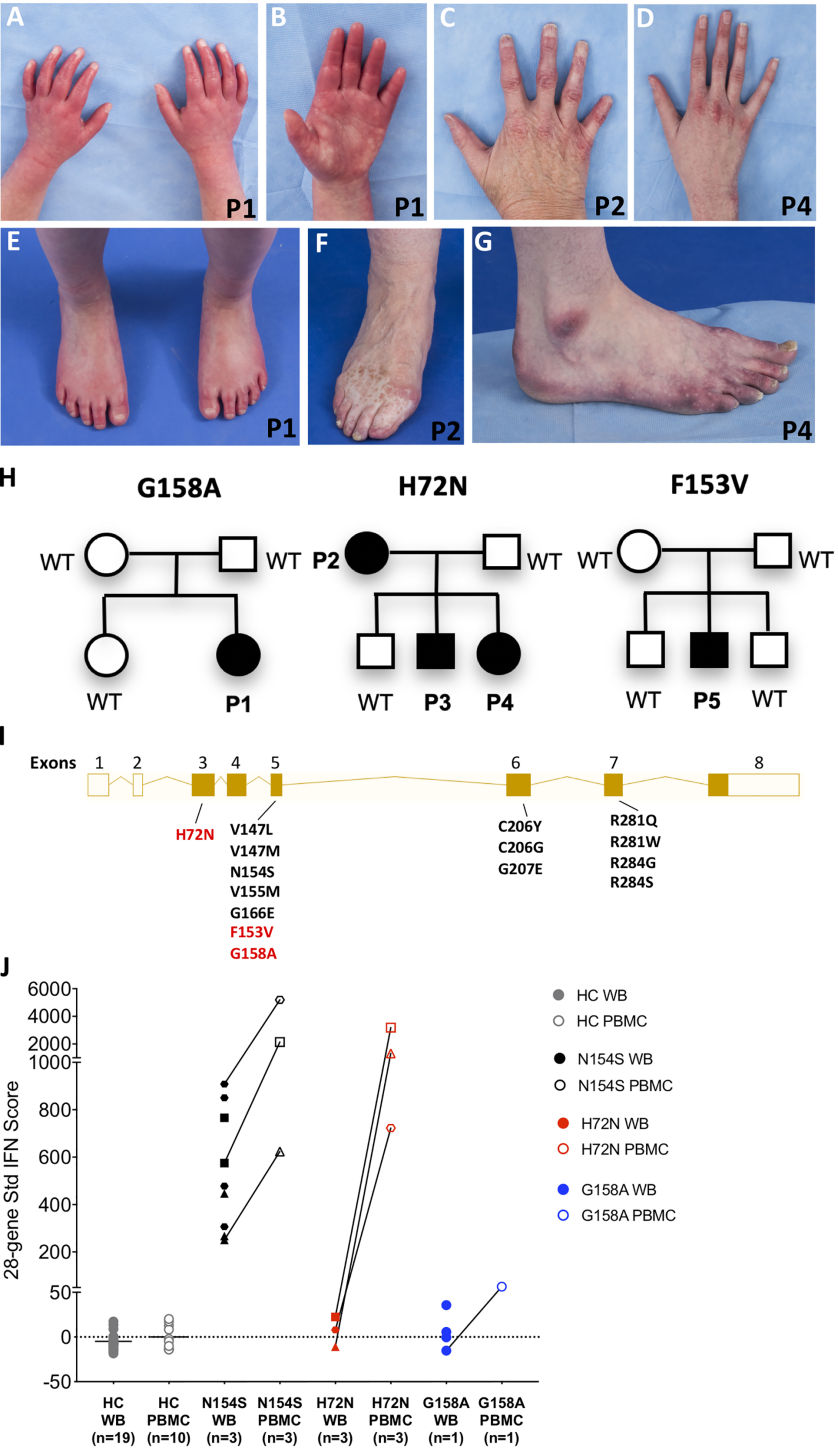
Three novel variants in STING1 cause SAVI. **(A–D)** Erythematous lesions of the hands and tapered fingers in Patient 1 **(A, B)**, Patient 2 **(C)** and Patient 4 **(D)**. **(E–G)** Erythematous rash on the feet and tapered toes in Patient 1 **(E)**, with similar lesions and tissue loss of the feet in Patient 2 **(F)** and Patient 4 **(G)**. Patient number is depicted on the bottom right corner of each picture. **(H)** Pedigree charts. Solid symbols represent affected individuals; open symbols, unaffected individuals. P1-P5: patients, WT: wild type. **(I)** Exon locations of all SAVI-causing STING1 variants. Open boxes represent non-coding exons, solid boxes represent coding exons, while lines represent introns. Previously reported variants are in black and the three novel variants in red. Transcript diagram was obtained from ENSEMBL (www.ensembl.org). **(J)** 28-gene Interferon (IFN) scores from whole blood (WB) and peripheral blood mononuclear cells (PBMCs). Solid symbols indicate WB and open symbols indicate PBMCs. Healthy control (HC) samples included 19 WB and 10 PBMCs. SAVI patients with N154S mutation (n=3) in black, family members with H72N mutation (n=3) in red, and patient with G158A mutation (n=1) in blue. Paired PBMC and WB IFN scores from the same time point are connected by a line. Each patient with SAVI is represented by a different symbol.

All patients presented with peripheral vasculopathy (**Figures 1A–G**), while lung involvement and features of systemic inflammation were more variable between patients. Not all of the patients received immunomodulatory treatment. Patients’ characteristics are summarized in **Table 1** and **Supplemental Table 1** and described below; family trees are shown in **Figure 1H**; exon locations of the mutations are shown in **Figure 1I**.

**Table 1:** Patient characteristics.

	Patient 1	Patient 2	Patient 3	Patient 4	Patient 5
**Gender**	F	F	M	F	M
**Ethnicity**	Caucasian	Caucasian	Caucasian	Caucasian	Caucasian
***STING1* mutation**	c.473G>C, p.G158A De novo	c.214 C>A, p.H72N -	c.214 C>A, p.H72N Inherited	c.214 C>A, p.H72N Inherited	c.457T>G, p.F153V De novo
**Age at onset**	12 months	4 years	3 months	2 months	1 year
**Age at last evaluation**	6 years and 7 months	54 years	21 years	19 years	28 years
**Recurrent fever**	+	–	–	–	+
**Failure to thrive**	+	–	–	–	–
**Increased inflammatory markers**	+	–	–	–	–
**Hematologic manifestations**	Neutropenia, leukopenia, anemia, thrombocytopenia	–	–	–	Lymphopenia
**Positive autoantibodies**	ANA, anticardiolipin IgG, anti-thyroid peroxidase	–	–	ANA	–
**Increased IFN score (whole blood)**	–	–	–	–	Not available
**Lung involvement**	Mild/Moderate	Mild	–	–	–
**Peripheral vasculopathy**	+	+	+	+	+
**Tissue loss**	Nasal septum perforation	–	–	–	–
**Arthritis**	–	–	–	–	–
**Basal ganglia calcifications**	Not available	–	Not available	–	Not available
**Previous treatment**	G-CSF, antibiotics	Sympathectomy	–	–	Diltiazem, nifedipine
**Current treatment**	Baricitinib	Amlodipine	Amlodipine	Amlodipine, Pentoxifylline	–

ANA, antinuclear antibodies; G-CSF, Granulocyte-colony stimulating factor.

Patient 1 is a 7-year-old girl of French-Canadian origin, who presented at 12 months of age with chronic rhinorrhea and recurrent fever. At 17 months, due to neutropenia, a bone marrow biopsy was performed, showing a hypocellular marrow (20%) with markedly decreased granulopoiesis, mildly decreased megakaryocytes, and mildly increased plasma cells. At the age of 22 months she developed persistent erythematous rashes involving hands, feet, buttocks, and cheeks that were aggravated by cold temperatures. At the age of 2 years she presented with chronic cough, growth retardation and speech delay. Due to persistent neutropenia, she was treated with G-CSF between the age of 27 months and 4 years. Whole Exome Sequencing (WES) at the age of 5 revealed a *de novo* heterozygous *STING1* variant, p.G158A, which is not observed in healthy populations from gnomAD database (https://gnomad.broadinstitute.org), and she was diagnosed with SAVI. She had red-purple lesions on hands and feet, tapered fingers without ulcers. Other features included livedo reticularis, telangiectasias on cold exposed areas, nasal septum perforation and a flat nasal bridge consistent with a saddle nose deformity (**Figures 1A, B, E**). Her weight was on the 8th percentile, height below the 1^st^ percentile with delayed bone age. A chest computed tomography (CT) showed ground glass opacities in the left lower lobe. Her pulmonary function tests (PFTs) showed mildly reduced spirometric lung volumes. She had an increased erythrocyte sedimentation rate (ESR; 44 mm/h), autoimmune hypothyroidism and strongly positive antinuclear antibodies (ANA; 4 EU, normal range 0-0.9 EU); anticardiolipin IgG was equivocal. Baricitinib was started at the age of 5 years and 10 months. Her peripheral vasculopathy remained stable, and on PFTs the forced vital capacity improved from 86% to 95% of the predicted value on baricitinib treatment. She developed mild macrocytic anemia (Hb >10 g/dl), thrombocytopenia (>100.000/μl), and neutropenia (>1.000/μl). At the last evaluation, after 9 months of baricitinib treatment, her ESR was still elevated (37 mm/h), anticardiolipin IgG was negative, while ANA remained positive albeit at lower titers (2.7 EU). Weight had reached the 39th percentile, height the 3^rd^ percentile.

Patient 2 is a 54-year-old Caucasian woman, with a history of chilblains and erythematous skin lesions over ears, nose, and cheeks since the age of 4. At the age of 6, she underwent sympathectomy and was started on calcium channel blockers which improved her symptoms. She also had a history of chronic upper respiratory congestion. Her right fifth finger was amputated after suspected post-traumatic osteomyelitis. Peripheral vasculopathy led to finger and toe tapering and dyschromic skin changes (**Figures 1C, F**). She never recalled fever, lymphadenopathy, hepatosplenomegaly, or respiratory symptoms. WES showed a heterozygous *STING1* variant, p.H72N, which is not observed in healthy populations from gnomAD database. Samples from her parents, who were reported to be healthy, were not available. A chest CT performed at the age of 54 showed reticular ground-glass opacities in the lower lobes, potentially representing scarring due to previous inflammation. Six-minute walking test (6MWT) and PFTs including diffusing capacity for carbon monoxide (DLCO) were normal. Inflammatory markers, complete blood count (CBC), lymphocyte subsets, and serum immunoglobulins were normal. Autoantibodies were negative. Height was normal, weight was above the 97^th^ percentile. She has two sons and one daughter, of whom two harbor the same *STING1* variant (Patient 3 and 4) and also developed SAVI as described below; her son without the mutation is healthy.

Patient 3 is a 21-year-old Caucasian man, who presented during infancy with cold induced skin color changes, and hypothermic fingers and toes with dry skin. He was started on amlodipine with good response. Patient 4 is a 19-year-old Caucasian woman, whose perinatal history was significant for cyanosis with normal oxygenation. At two months of age she presented with chilblains involving feet, hands, nose and ears (**Figures 1D, G**). Cold would also cause numbness in feet, while hot temperatures would worsen rash and pain. She was started on amlodipine with good response and pentoxyphylline was later added. She had a history of a phonological speech disorder and chronic upper respiratory congestion. Both siblings did not develop ulcerations or tissue loss, fever, arthritis, lymphadenopathy, hepatosplenomegaly, or respiratory symptoms. Chest CT, 6MWT and PFTs including DLCO performed at the ages of 21 and 19 respectively were normal. Inflammatory markers, CBC, lymphocyte subsets, and serum immunoglobulins were normal. Autoantibodies including ANA were negative for the brother, but ANA was positive in the sister (2.2 EU). Both siblings had normal height and weight.

Patient 5 is a 28-year-old Caucasian man, who presented from the age of one year with telangiectasias and chilblains lesions of nose, cheeks, hands and feet during winter months. Capillaroscopy revealed capillary dilation and tortuosity. Episodes of recurrent fevers started at age 26 without apparent triggers. His neurodevelopment was normal. He never developed failure to thrive, and denied arthritis, lymphadenopathy, hepatosplenomegaly, or respiratory symptoms. A chest CT performed at age 27 was normal, a CBC revealed lymphopenia. Inflammatory markers were normal and autoantibodies (including ANA, anti-dsDNA, antibodies to extractable nuclear antigens and antineutrophil cytoplasmic antibodies) were negative, serum immunoglobulins were normal. Genetic testing showed a heterozygous *STING1* variant. p.F153V, which is not observed in healthy populations from gnomAD database. He is treated with calcium channel blockers.

### Interferon Signature in Mononuclear Cells but Not Whole Blood

Most SAVI patients present with an elevated interferon response gene signature recognized as elevated interferon scores (14). As expected, interferon scores from peripheral blood mononuclear cell (PBMC) samples of patient 1-4 are much higher than those of healthy controls (**Figure 1J**); samples from patient 5 were not available. Notably, interferon scores from the whole blood samples were negative, which is different from previously reported SAVI mutants including p.N154S (**Figure 1J**). The mechanism remains unclear. Further investigation is needed to assess the interferon scores in various cell populations of the blood, and in patients with the same mutation from different kindreds that may become available in the future.

### p.H72N, p.G158A, p.F153V Variants Lead to STING Autoactivation

**Figure 2:**
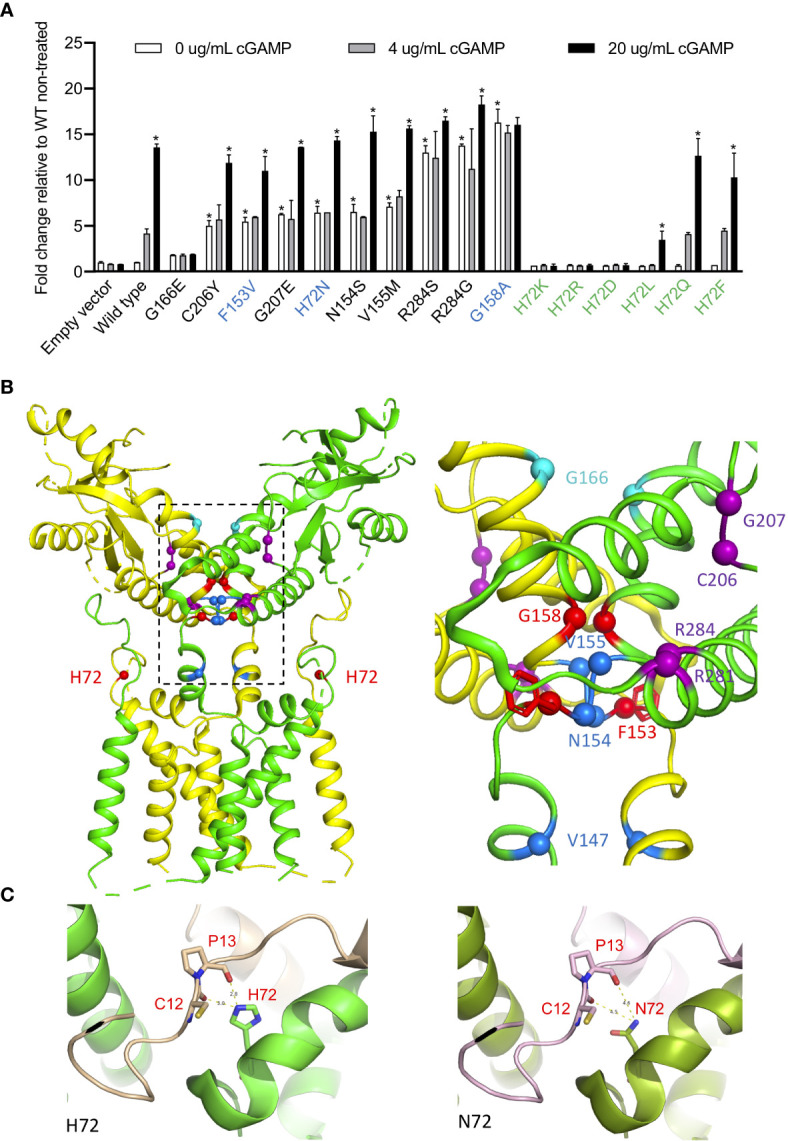
H72N, G158A, F153V variants lead to autoactivation. **(A)** Novel STING variants lead to strong STING autoactivation as measured by *IFNB1* reporter activation in 293T cells. 50 pg of various STING constructs were transfected. The three novel SAVI-causing variants are labeled in blue, and other H72 variants are labeled in green. The SAVI-causing variants are shown in the order of autoactivating potential. *An *asterisk* above the *white bar* designating 0 μg/ml cGAMP indicates significant change compared (p<0.0001) to wildtype (WT) non-treated, by ordinary one-way ANOVA Dunnett’s multiple comparisons tests. An *asterisk* above the *black bar* designating 20 μg/ml cGAMP indicates significant change (p<0.003) of 20 μg/ml cGAMP treated cells compared to the respective non-treated cells; by two-way ANOVA, Bonferroni’s multiple comparisons test. Data are presented as mean±SD of duplicates. Similar results were obtained in independent experiments. **(B)** Ribbon structural model of apo STING dimer, with the two subunits colored in yellow and green. Location of SAVI-causing variants are highlighted with spheres, with the three novel variants in red, variants in the connector helix loop region in blue, p.G166E variants in the ligand-binding pocket in cyan, and variants in the polymer interface in purple. The major mutation region is enlarged on the right, showing how the F153 phenyl ring buttress the polymer interface. **(C)** Structure model of H72 compared to N72 indicates increased dimer flexibility of p.H72N mutation, which confers gain-of-function. The two STING monomers forming a dimer are in green and gold/purple respectively.

To assess the autoactivating potential of the 3 novel variants, constructs expressing mutant or wild type (WT) STING were transfected into HEK293T cells and *IFNB1* firefly luciferase reporter activation was measured (**Figure 2A**). All 3 mutant constructs showed autoactivation in the absence of ligand (**Figure 2A**). The autoactivation was not due to higher expression of the mutant proteins compared to the WT (**Supplementary Figure S1**). The p.F153V and p.H72N mutations remained responsive to ligand stimulation with cGAMP, while the p.G158A mutant was maximally activated at baseline and was not further enhanced by cGAMP stimulation. The constitutive auto-activation of p.G158A was even higher than WT construct activated with 20 µg/mL of cGAMP ligand (**Figure 2A**).

The p.G158A mutation in the connector helix loop increased STING activation more than 13-fold, which is comparable to two other “highly-autoactivating” mutations, p.R284S and p.R284G, in the polymer interface (**Figure 2A**, see also **Figure 2B** for the residue location). Their activation mechanism differs and was previously described by different models (12, 15). The p.G158A mutation is predicted by a rotation model to highly mimic the ligand-bound form of STING thus conferring autoactivation (12). The model suggests that cGAMP binding on wildtype STING triggers a 180° rotation of the ligand binding “head” of the STING dimer, which allows a closed dimer conformation, “flattening” of the polymer interface and STING polymerization *via* side-by-side packing (12). The STING oligomers recruit TBK1 and IRF3 which activate downstream signaling (16). Consistent with this model, the p.G158 residue that is mutated in patient 1 is situated in a closely packed location of the apo STING dimer, where only Glycine, the smallest residue, can be accommodated (12) (**Figure 2B**). Mutation to the larger Alanine results in “space limitation” that is predicted by the model to “force” a 180° rotation of the ligand-binding head, which mimics the ligand-bound STING conformation thus conferring autoactivation.

The p.R284S and p.R284G highly-autoactivating mutations, however, are not likely to trigger a rotation and are better explained by a polymer interface blocking model (15). This model suggests that STING is kept inactive *via* C-terminal tail binding to the polymer interface, which blocks polymerization and prevents autoactivation. As a result, mutating the polymer interface residues to various residues lead to broad autoactivation (13, 15). However, neither model explains STING autoactivation caused by the p.H72N and p.F153V mutations; both are unlikely to “push” a 180° rotation and are not located in the polymer interface.

### The Variant p.H72N Identifies a Novel Class of SAVI-Causing Mutations That Are Located in the Transmembrane Linker Domain

While the previously reported SAVI-causing mutations are distributed around the dimer crossing-over region, the disease-causing p.H72N variant identified in patients 2-4 is the first SAVI-causing mutation in the transmembrane linker region of STING (**Figure 2B**) that, together with the N-terminal tail, forms a supporting arm which restrains the ligand-binding domain (12) (**Figure 2B**). However, neighboring mutations in the transmembrane linker region, p.E68A and p.E69A, resulted in loss-of-function (12) which raised the question how the p.H72N mutation would confer a gain-of-function.

To interrogate how residue p.H72 might control STING activation, we generated various mutant constructs at the p.H72 position and assessed their autoactivating potential and their response to cGAMP stimulation in activating interferon activity (**Figure 2A**). Mutations to charged residues, including Lysine (K), Arginine (R), or Aspartic acid (D), led to a complete loss-of-function with no autoactivation and loss of a cGAMP response (**Figure 2A**). Mutations to the hydrophobic residue Leucine (L) resulted in a dramatic reduction in the cGAMP response. In contrast, mutations to residues with uncharged polar side chains, including Glutamine (Q) or Phenylalanine (F), resulted in responses similar to wildtype construct, with a maintained dose dependent cGAMP response. These data suggest that the charged and hydrophobic residues may result in improper STING dimer formation, while residues with uncharged polar side chains may maintain the wildtype configuration of the dimer.

### Structural Modeling of p.H72N Suggests a Refined Model of STING Activation

We hypothesized that residues with uncharged polar side chains at position p.H72 may maintain the “supporting arm” structure to restrain the ligand-binding head, and we generated structural models to investigate the effect of the mutation on its interactions with neighboring residues (**Figure 2C**). Structural modeling of the domain in fact confirms that residue p.H72 is essential in forming hydrogen bonds with residues 12 and 13 on the N-terminal tail of the second STING monomer within the STING dimer (**Figure 2C**, left), which stabilizes a “supporting arm”. If mutated to Asparagine (N), these hydrogen bonds cannot form firmly (**Figure 2C**, right), which would result in a “looser support” of the ligand binding head and increased head flexibility. The polymer interface, which is on the outer surface of the ligand binding head, would also become more flexible, which attenuates its binding to the C-terminal tail (15) thus conferring the autoactivation.

These findings highlight an essential role of hydrogen bonds at the p.H72 location: consistent with our observations, only polar residues can stabilize the supporting arm *via* hydrogen bonds, while both charged and hydrophobic residues fail to form hydrogen bonds and results in loss-of-function (**Figure 2A**). The autoactivation *via* a 180° rotation is energy consuming and although likely favored by the p.G158A mutation that would “force” the rotation due to space limitations, it is not a likely mechanism in patients with the p.H72N mutation, which may only allow random rotations that are inefficient in causing autoactivation.

In fact, rotation of the ligand-binding head is also an unlikely consequence of the highly-autoactivating polymer interface mutations (13) (**Figure 2A**), as these mutations are located in the dimer surface and should not cause a rotation of the ligand-biding head (**Figures 2A, B**, purple labeled). These findings are in concordance with a structural study of bacterial cyclic-di-GMP (CDG)-bound STING, which revealed that CDG can activate STING without a rotation and a STING dimer closing (15). Furthermore, STING binding to TBK1 is quite flexible through its unordered C-terminal tail, which was predicted to occur even in an open dimer conformation (16).

A presumed “small protrusion” in the polymer interface of unrotated STING was thought to be sufficient to keep STING inactive (12). However, the aforementioned examples of STING activation in an “unrotated state” (with the protrusion still present) raised questions about the exclusive validity of that model and led to the proposal of an inhibitor that blocked STING polymerization (13). In fact, the C-terminal tail binding can serve that function (15) and no other inhibitor protein is needed, as autoinhibitory STING dimers can be formed in a cryo-EM structure where only STING is present (12).

**Figure 3:**
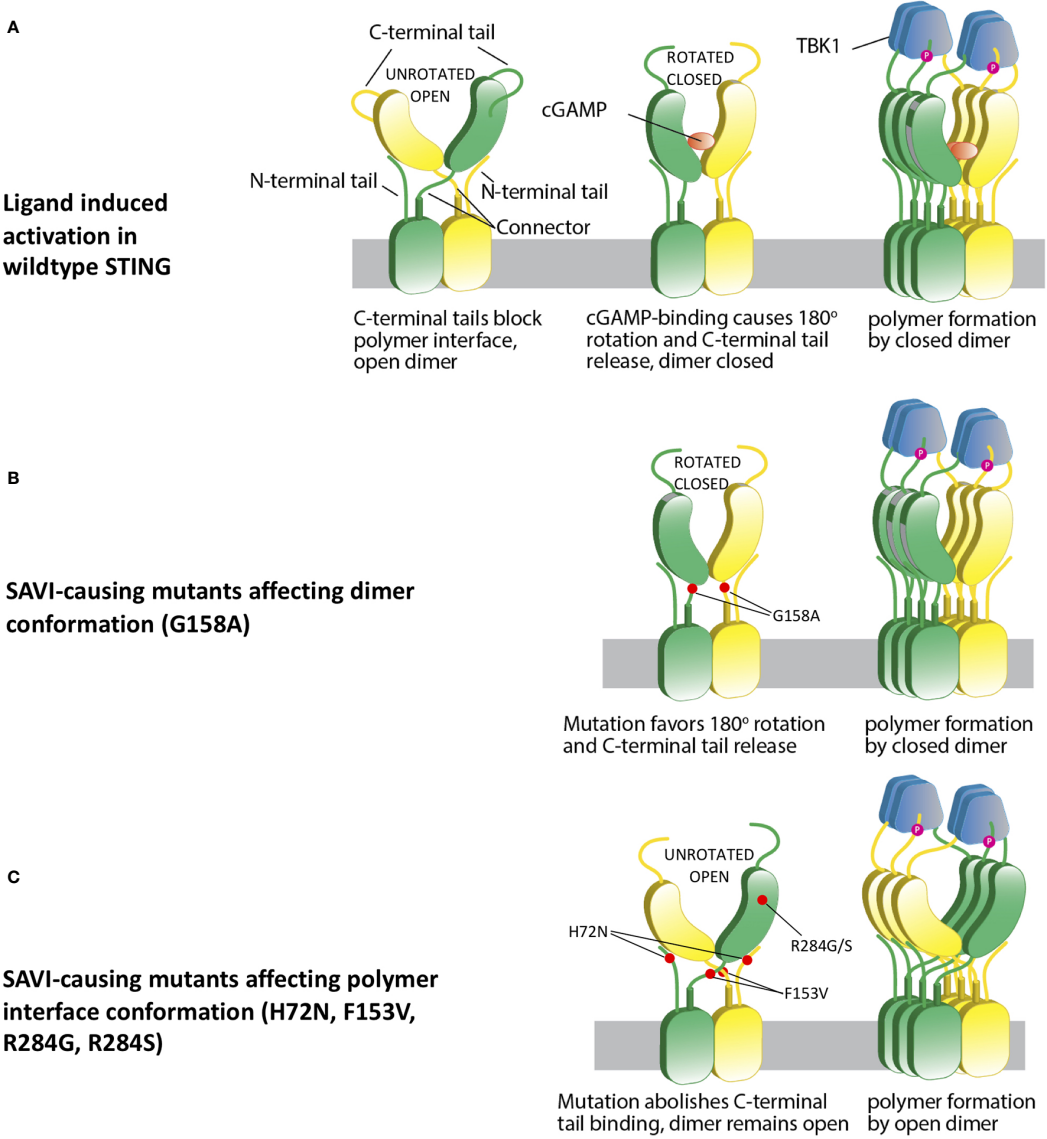
A hypothetical STING activation model reconciles current structural models. **(A)** Wild type apo STING forms a dimer in open conformation, with C-terminal tail blocking the polymer interface and preventing polymerization. cGAMP binding leads to a 180° rotation and a closed dimer, which also changes the polymer interface conformation and subsequently loses C-terminal tail blocking. This leads to STING polymerization *via* side-by-side packing and TBK1 recruitment. **(B)** SAVI mutant G158A “pushes” the formation of a closed dimer, which mimics ligand-bound STING and confers autoactivation in the absence of ligand binding. **(C)** Some other SAVI mutants like R284G, R284S, H72N, F153V directly affect the polymer interface conformation, which leads to release of C-terminal tail binding. The STING dimer remains in the open state, but the polymer interfaces are free of C-terminal tail blocking, which allows polymerization and autoactivation.

These observations led to the development of an adjusted model of STING activation that accommodates all currently reported STING-causing mutations in exons 3, 5, 6 and 7 (13). We hypothesize that STING is kept inactive by the C-terminal tail blocking of the polymer interface, which prevents polymerization *via* side-by-side packing (**Figure 3A**). Ligand binding induces 180° rotation of the ligand binding domain and leads to conformational change in the polymer interface, which results in loss of C-terminal tail binding. This clears the polymer interface and allows polymer formation *via* side-by-side packing (**Figure 3A**).

In this reconciled model, SAVI-causing mutations can be categorized into 4 classes. The p.G158A variant and possibly the p.N154S and p.V155M variants, which are all located in the tightly packed connector region, mimic the ligand-bound STING dimer by favoring a “rotated” conformation (**Figure 3B**). Mutations in the polymer interface at residues R281, R284, C206, and G207, constitute the second class, which directly abolishes binding to the autoinhibitory C-terminal tail and allows side-by-side packing without a 180° rotation (**Figure 3C**); p.H72N, p.F153V, p.V147L, and p.V147M constitute a third class which relieve the constraint on the polymer interface and autoactivate without a rotation. Residues H72 and V147 are part of the supporting arm which stabilizes the ligand-binding head and preserves the C-terminal tail binding to the polymer interface. Residue p.F153 lies right beneath the polymer interface to constrain it and prevents STING polymerization (12) (**Figure 2B**). A residue with reduced size such as the phenylalanine (F) to Valine (V) (p.F153V) mutation would be predicted to increase the flexibility of the polymer interface, thus relieving the autoinhibition by loosening the C-terminal tail binding. The p.G166E located in the ligand binding pocket constitutes class 4. This mutation confers a very low level of autoactivation and also abolishes cGAMP responsiveness (**Figure 2A**). Its autoactivating potential is not explained by current models, and it is not clear whether this low autoactivation causes SAVI. It is possible that binding of a non-cGAMP ligand is needed to activate STING and cause the disease.

### Novel Mutations Are Associated With Milder Disease

Patients with mutations that were of lower auto-activating potential p.G166E, p.F153V, p.H72N (**Figure 2A**), showed milder disease with less severe or no lung manifestations, which is in contrast to the p.N154S, p.V155M mutations. However, the patient with the highly-autoactivating mutation, p.G158A, showed an intermediate phenotype, less severe than that of patients who develop interstitial lung disease and peripheral tissue loss including autoamputations. Her clinical picture was characterized by peripheral vasculopathy with nasal septal perforation, mild to moderate lung involvement, and hematologic manifestations (neutropenia). These findings indicate that disease severity in SAVI is influenced by additional factors that include family genetic backgrounds, treatment histories and yet unknown environmental factors such as infections which all may contribute to the variable disease severity which is even seen within the same family (6). Additional studies of patients from different families with the same mutations would help to address this question.

## Discussion

We have identified three novel SAVI-causing mutations in critical locations of STING. All patients had more prominent peripheral disease with mild or no lung disease. The p.H72N mutation is the first SAVI-causing mutation discovered in the transmembrane linker region, and the other two mutations, p.F153V and p.G158A, had been predicted to be autoactivating by Cryo-EM structures (12). The typical clinical phenotype and the autoactivation of these mutations in the *IFNB1* luciferase reporter assay coupled with the prediction of the autoactivating potential of these mutations in Cryo-EM structural studies and their absence in publicly available databases including gnomAD database confirms that they are disease-causing. Future identification of more patients from different families would help better address the variable disease severity caused by the respective mutations. Future studies of interferon-dependent and -independent functions of STING that may contribute to the disease phenotype are also planned (17–21).

The p.H72N and p.F153V mutations, however, are not fully explained by a model that requires rotation of the ligand-binding head for downstream activation (12), as both are unlikely to force a rotation and form a closed dimer. This is similar to the highly-autoactivating mutations in the polymer interface, which were explained by another model (15). Our data suggest a reconciled model that allows both open and closed STING dimers to form polymers and lead to STING activation, as long as the polymer interface is cleared of the C-terminal tail binding which blocks oligomerization of the STING dimers.

Although our findings are limited by our inability to generate a cryo-EM model, which was outside the scope of our work, these findings have important implications for the use and design of drugs that target STING for the treatment of SAVI patients and likely other diseases that may activate the cGAS-STING pathway. As described in **Figure 3**, successful polymerization is the most critical step for autoactivation and is required by all SAVI-causing mutations, suggesting that the polymer interface should be the preferred drug target site to attenuate STING autoactivation.

We have recommended JAK inhibitors for SAVI patients and have reported on their partial responses, particularly on the stabilization of the lung disease (22). Interestingly, Patients 1 and 4 were treated with baricitinib for 2-3 months with only marginal benefit to their peripheral vascular disease, and both discontinued treatments. Thus, the risks and benefits in patients with mild disease have to be considered carefully.

In summary, the novel mutations expand the phenotypic presentation of SAVI by including clinical presentations with mild or no lung involvement and propose novel mechanisms of STING activation. Our findings led to a refined model of STING activation, which will inform the development of drugs that target STING activation in patients with SAVI and other STING-dependent inflammatory diseases.

## Data Availability Statement

The original contributions presented in the study are included in the article/**Supplementary Material**, further inquiries can be directed to the corresponding authors.

## Ethics Statement

The studies involving human participants were reviewed and approved by Institutional Review Board of National Institutes of Health. Written informed consent to participate in this study was provided by the participants or their legal guardian/next of kin. Written informed consent was obtained from the individual(s), and minor(s)’ legal guardian/next of kin, for the publication of any potentially identifiable images or data included in this article.

## Author Contributions

Conceptualization: BL, AJ, and RG-M. Experiments: DK, BL, JM, AJ, and AA. Patient data collection: ST, DR, BW, MM-C, LS, SA, AJ, and RG-M. Structural modeling: ZJ and TJ. Writing of initial draft: BL, ST, and AJ. Writing – review and editing: BL and RG-M. All authors contributed to the article and approved the submitted version.

## Funding

This research was supported by the Intramural Research Program of NIAID, NIH.

## Conflict of Interest

RG-M has received grant support from SOBI, Regeneron, Novartis and Eli Lilly.

The remaining authors declare that the research was conducted in the absence of any commercial or financial relationships that could be construed as a potential conflict of interest.

## Abbreviations

*STING1*, Stimulator of interferon response cGAMP interactor 1; SAVI, STING-associated vasculopathy with onset in infancy; IFN, Interferon; cGAMP, 2’3’-cyclic GMP–AMP; WES, whole exome sequencing.
