# Supplementary material for: Case Report: Novel SAVI-Causing Variants in STING1 Expand the Clinical Disease Spectrum and Suggest a Refined Model of STING Activation
Source: Front Immunol. 2021 Mar 22;12:636225. doi: 10.3389/fimmu.2021.636225 (PMC8023226; doi:10.3389/fimmu.2021.636225)
Supplement: Supplementary file 1 [file DataSheet_1.docx]

Supplementary Material

**Novel SAVI-causing variants in *STING1* expand the clinical disease spectrum and suggest a refined model of STING activation**

**Supplementary Figure**

**Supplementary Figure 1. Protein expression of transfected constructs in HEK293T cells by Western blot.** 800 pg of different constructs were transfected into 120,000 HEK293T cells and STING and ACTB were detected in chemiluminescent and Rhodamine channels respectively in the same blot.

**Supplementary Table 1. Blood cell count of patients**

|  | **Patient 1** | **Normal range** | **Patient 1** | **Normal range** | **Patient 1** | **Normal range** | **Patient 1** | **Normal range** | **Patient 2** | **Normal range** | **Patient 3** | **Normal range** | **Patient 4** | **Normal range** | **Patient 5** | **Normal range** |
| --- | --- | --- | --- | --- | --- | --- | --- | --- | --- | --- | --- | --- | --- | --- | --- | --- |
| **Age at exam (years)** | 1.4 | | 4.3 | | 5.3 | | 6.5 | | 54 | | 21 | | 19 | | 27 | |
| **Hemoglobin (g/dL)** | 8.2 | 10.5-13.5 | 11.4 | 11.5-13.5 | 12.5 | 10.2-12.7 | 10.1 | 10.6-13.2 | 14.3 | 11.2-15.7 | 14.9 | 13.7-17.5 | 13.7 | 11.2-15.7 | 15.4 | 13.6-18.0 |
| **Platelets (10^3^/uL)** | 109 | 140-450 | 248 | 140-450 | 220 | 189-394 | 133 | 199-367 | 249 | 173-369 | 217 | 161-347 | 264 | 173-369 | 270 | 130-440 |
| **WBC (cells/uL)** | 2300 | 6000-17500 | 10200 | 486-13180 | 7420 | 4860-13180 | 6840 | 4270-11400 | 6840 | 3980-10040 | 5120 | 4230-9070 | 7450 | 3980-10040 | 6470 | 3800-11500 |
| **ANC (cells/uL)** | <100 | 1500-8500 | 4100 | 1500-8500 | 2810 | 1600-8290 | 1340 | 1640-7870 | 3910 | 1560-6130 | 2810 | 1780-5380 | 4250 | 1560-6130 | 4880 | 2500-7500 |
| **ALC (cells/uL)** | 2300 | 4000-10500 | 5000 | 1250-5770 | 4270 | 1250-5770 | 3830 | 1160-4280 | 2080 | 1180-3740 | 1620 | 1320-3570 | 2100 | 1180-3740 | 1600 | 1500-4000 |
| **CD3+ lymphocytes** | n.a. |  | n.a. |  | 3638 | 1400 - 3700 | 3033 | 1200 - 2600 | 1348 | 714-2266 | 1225 | 714-2266 | 1747 | 714-2266 | n.a. |  |
| **CD3+CD4+ lymphocytes** | n.a. |  | n.a. |  | 1524 | 700 - 2200 | 1191 | 650-1500 | 1061 | 359-1565 | 816 | 359-1565 | 1096 | 359-1565 | n.a. |  |
| **CD3+CD8+ lymphocytes** | n.a. |  | n.a. |  | 2079 | 490 - 1300 | 1827 | 560-1700 | 268 | 178-853 | 382 | 178-853 | 601 | 178-853 | n.a. |  |
| **CD19+ lymphocytes** | n.a. |  | n.a. |  | 474 | 390 - 1400 | 666 | 270 - 860 | 258 | 61-321 | 227 | 61-321 | 202 | 61-321 | n.a. |  |
| **NK cells** | n.a. |  | n.a. |  | 132 | 130 - 720 | 107 | 100 - 480 | 453 | 126-729 | 156 | 126-729 | 126 | 126-729 | n.a. |  |
| **Monocytes (cells/uL)** | 0 | 0-1000 | 900 | 0-1000 | 330 | 24-920 | 220 | 190-810 | 620 | 240-860 | 570 | 300-820 | 820 | 240-860 | 390 | 200-800 |
| **CRP (mg/L)** | n.a. |  | n.a. |  | 2.8 | 0-4.99 |  | 0-4.99 | 3.7 | 0-4.99 | 1 | 0-4.99 | 0.5 | 0-4.99 | 0.7 | 0-5.0 |
| **ESR (mm/h)** | n.a. |  | n.a. |  | 44 | 0-20 | 37 | 0-20 | 7 | 0-20 | 2 | 0-20 | 4 | 0-20 | 3 | 1-12 |

ALC: Absolute Lymphocyte Count. ANC: Absolute Neutrophil Count. CRP: C-reactive protein. ESR: Erythrocyte Sedimentation Rate. n.a.: not available. WBC: White Blood Cells. Patient 1 was treated with G-CSF between the age of 2 years and 3 months and the age of 4 years; she was treated with baricitinib since the age of 5 years and 10 months.
